# Breakers Ahead

**Published:** 1892-02-13

**Authors:** 


### PASSING TOPICS.

### BREAKERS AHEAD.

When the Lord Chancellor observed at the recent meeting of the Hospitals Association, that he was appalled at the comparatively small number of persons who help to maintain our hospitals, it was evident that a fact only too well known to hospital workers for many years past had come to him in the way of a revelation. We hope Lord Halsbury's observation will strike home to the consciences of a great many of the people hitherto content to accept the platitudes always plentiful in regard to the system of voluntary support, and to do nothing either by hand or purse to help forward those great and indispensible institutions of which we have been accustomed to be proud, though now, if we are to believe some of their critics, it is nothing short of a misdemeanour to speak well.

Lord Halsbury at any rate does not hesitate as to the nature of his testimony. Whether conscious of shortcomings or not, he is just enough to perceive that there is a balance of merit much too great to be made away with. If we take exception to anything his lordship said it is to the genial supposition that nothing but a wider knowledge of hospital wants is needed to ensure that they shall be supplied. We gladly endorse the dictum that inattention "is not due to want of heart but want of thought" on the part of the public, but it is difficult to understand how anybody who reads a newspaper, or dips into current literature of any sort, or who possesses a letter box into which appeals can be dropped, fails to be unacquainted with the desperate straits of the hospitals, or of the continuous and almost frantic efforts put forward by one and another of the institutions to attain to a financial equilibrium.

Possibly the attention which the cunningest devices of hospital managers and secretaries have failed to obtain may be more readily conceded to Lord Halsbury's utterances. Nothing could better reward his lordship's efforts than the knowledge that a fuller recognition of hospital necessities will date from them. Individual hospitals have never lacked distinguished advocates, who have made themselves heard at festivals and other gatherings, but when a Lord Chancellor presides over a meeting called together to hear something about the chronic poverty of our hospitals as a body, there is a certain novelty in the proceedings, calculated, as we hope, to render them of more than passing interest and of good augury to the cause which the people of all classes, were they but masters of its importance, would hasten to make their own.

These are critical times for the hospitals; how critical, only those who read them attentively can realise, and nothing but disaster can result from a refusal on the part of hospital friends and supporters to look facts of all sorts full in the face. Already the radical section of candidates for the London County Council are proposing to add the control of the hospitals to the bill of fare in preparation for their omnivorous maw, and if we would save our institutions from a fate like that, or what is akin to it, we must be prepared to do something more than sit still and complain of adverse seasons to the moon.

Agricola.

